# Qualitative and Quantitative Detection of Mealworm DNA in Raw and Commercial Food Products Using Real-Time PCR

**DOI:** 10.3390/genes13081400

**Published:** 2022-08-06

**Authors:** Małgorzata Natonek-Wiśniewska, Piotr Krzyścin, Anna Koseniuk

**Affiliations:** Department of Animal Molecular Biology, National Research Institute of Animal Production, 1, Krakowska Street, 32-083 Balice, Poland

**Keywords:** insects, mealworm, species identification, mtDNA

## Abstract

Considering food safety and an increasing public awareness of the ingredients, production process and origin of foods, the application of insects as food requires the development of tests for the reliable identification of their presence. The aim of the study was (1) the determination of appropriate modifications of the selected method for isolating the DNA of two life stages of mealworm, i.e., larva and adult, from commercial food products; (2) the determination of the method parameters for the qualitative and quantitative analysis of mealworm contents based on the detection of a species-specific mitochondrial DNA fragment, using real-time PCR; (3) the application of a method to test the commercial food products of mealworm. A total of nine species of adult insect were investigated (field cricket, Dubia cockroach, Madagascar cockroach, banded cricket, migratory locust, yellow mealworm, superworm, house fly and lacewing), theirlarvaes (yellow mealworms and superworms) and thirteen commercial food products (dried whole insects, powder and granules) representing various insect species and origins which were purchased from the European market. The obtained results showed that the efficiency of the modification of the DNA extraction method is dependent on the life stage of the mealworm. We proved the high sensitivity of the test, with the range of the method being 0.1–100%; we also proved the biological specificity in this range, and the linearity. The linearity of the test was also statistically verified using the Fisher–Snedecor test. One-way variance analysis showed statistically significant differences between the c_T_ values of the two mealworm life stages studied, and similarly, between the threshold cycle (c_T_) values of adult forms. In contrast, for the inside group of mealworm larvae, there was no significant difference observed between the results of the c_T_ values. The test is effective for processed food products and may be used to monitor food. The research proved the suitability of the applied method for the analysis of samples that are commercially available as food for exotic animals. The hereby-developed method is based on widely used laboratory techniques, and does not require any additional investment in equipment. The availabilityof such a methodallows for the verification of the accuracy of the declared species component of the food products.

## 1. Introduction

The use of insects as a source of food is thought to be as ancient as humanity. However, for many centuries, it has been slightly forgotten, and it is limited territorially mainly to Asia, Africa and South America. In European countries, the consumption of insects has negative cultural connotations and is considered repulsive. For this reason, research into this issue has been neglected for many years [1]. It is only recently that there has been renewed interest in insects as a diet ingredient for both animals and humans.

The International Feed Industry Federation (IFIF) reported that the world population will reach more than 10 billion people by 2050 [2], and they will probably consume almost double the amount of animal protein [3]. Part of the animal protein can be delivered directly from insects, but a second part can be delivered from animals that have been fed with insects. This means that insects will be a critical component of the integrated food chain in the future [4].

Moreover, according to a report by the Food and Agriculture Organisation of the United Nations, insects at different stages of development are a promising source of food for biological, environmental and economic reasons. Insects are considered an optimal source of food because of their high protein content, well-balanced amino acid profile and abundance in fat and vitamins [5,6,7,8,9].

These advantages, along with low ammonia emissions during insects’ life cycles [10] and the low rearing costs, make them a promising source of protein in industrial feed production, which is now the subject of intensive research in many countries [11]. As a result of these studies, insects have been allowed to be used in animal feed. In 2017, the European Commission endorsed the use of insects to feed farmed fish [12]. In addition, the same European Commission document confirms that insects may provide a lasting alternative to conventional sources of animal protein intended as processed animal proteins for farm animals other than ruminants.

The emergence of these regulations creates a need for ready-to-use analytical tools and the implementation of an appropriate control system [13]. It is essential to develop laboratory methods that will be useful for monitoring the identification and prevention of food fraud cases [14]. In the last two decades, the species composition of food products has been mainly authenticated via molecular analysis, because DNA is identical in all somatic cells of an organism, and remains unchanged, regardless of the source of origin (blood, muscles, etc.). Furthermore, because researchers use degradation-resistant DNA fragments, the analyses are effective for highly processed food products and trace contaminants. These methods are highly sensitive, because the amount of material required for efficient detection may be just a few cells [15]. The most common techniques used to analyse species components in food include conventional and real-time polymerase chain reaction (PCR), PCR single-strand conformation polymorphism, and random amplified polymorphic DNA [16]. In addition, the European Union (EU) allows only light microscopy and PCR for the analysis of processed animal proteins [17].

The list of insects, the identification of which is of particular interest to scientists, is limited to the following seven species recommended for feeding purposes by the EU Regulation [12]: the black soldier fly (*Hermetiaillucens*) and house fly (*Musca domestica*), yellow mealworm (*Tenebrio molitor*) and lesser mealworm (*Alphitobiusdiaperinus*), house cricket (*Acheta domesticus*), banded cricket (*Gryllodessigillatus*) and field cricket (*Gryllusassimilis*).

The molecular insect identification methods presented in the literature are based on the analysis of a mitochondrial DNA (mtDNA) fragment that is biologically specific to the analysed species (barcoding DNA). As the insect class is enormous, detecting a fragment that will be specific to only one species is a complex challenge. To date, the species identification of insects has been limited for only the qualitative analysis of a few species intended for animal feed. For example, Marien et al. [18] reported a primer sequence for the detection of *H. illucens*, and Debode et al. [19] reported one for the detection of *T. molitor*.

Our topic of interest is the primers designated by Debode. On their basis we wanted to:

(1) Develop an efficient test for the qualitative and quantitative determination of yellow mealworms, based on the detection of a cytochrome I oxidase (mtDNA) fragment- specific species studied;
(2) Validate the test and statistically confirm the qualitative and quantitative identification of mealworms;
(3) Apply the developed test for the analysis of commercial food products available in the European market.

Moreover, in order to develop a complete mealworm identification test, we decided to:

(4) Determinate a DNA isolation method appropriate for raw samples of two life stages for this insect (adults and larvae) and commercial food products.

## 2. Materials and Methods

A total of nine species of adult insect (12 insects from each species) were investigated: field cricket (*G. assimilis*), Dubia cockroach (*Blapticadubia*), Madagascar cockroach (*Gromphadorhinaportentosa*), banded cricket (*G. sigillatus*), migratory locust (*Locusta migratoria*), yellow mealworm (*T. molitor*), superworm (*Zophobasmorio*), house fly (*M. domestica*) (two insects) and lacewing (*Chrysoperlacarnea*) (two insects).

The larvae (around 100 insects of each species) of yellow mealworms and superworms were purchased from breeders of feeder insects, which were reared under the Veterinary Inspectorate’s supervision to ensure that the material conformed to the declaration. The species of the studied material were confirmed via a comparison with photographs [20]. All insects were washed with distilled water and immediately stored in a freezer at −20 °C until use. All plant (lemon, banana, tomato, wheat grain, and oat grain) and meat (cattle, pig, turkey, chicken and fish) samples were purchased in a grocery store. Test samples of 0.5 g were prepared from each insect species. The number of repeats is described in the text and shown in tables.

Thirteen commercial food products representing various insect species, product types and origins were purchased from the European market. The samples, including dried whole insects (*n* = 8), powder (*n* = 4) and granules (*n* = 2) were immediately stored at room temperature until analysis.

Each raw sample was ground in liquid nitrogen to obtain DNA with good purity and yield. The commercial food products was ground at room temperature.

A detailed description of the test material is presented in Table 1. Repeated samples of the same species came from different batches of insects. Each batch was purchased at a different time.

### 2.1. DNA Isolation

In the first part of the study, the best method of DNA isolation was selected. For this purpose, selected samples of the yellow mealworms and superworms were isolated using the Sherlock kit (A&A Biotechnology, Poland) with dithiothreitol (DTT) according to the manufacturer’s instructions, with several modifications (I–IV):

(I) Isolation from higher than the manufacturer’s recommended sample weight (0.5 g) with a longer incubation time (4 h) and intense vortexing at 30-min intervals. 

(II) Isolation from three sample repeats (according to modification I), pooling of three filtrates on a filtration column, and further DNA isolation as one sample.

(III) Isolation of the insect sample (0.5 g) after washing twice with nuclease-free water (400 μL) through intense vortexing and centrifugation (5000 rpm/1 min). The combined supernatants were centrifuged (10 min, 13,000× *g*), the resulting pellet was washed with ddH2O (400 μL), and DNA was isolated.

(IV) DNA isolation from three sample repeats, was performed similarly to the previous modification, except that the filtrates obtained from the filtration column are pooled together on a purification column, and the DNA is extracted as one sample. The DNA from the other insect species was then extracted using the most effective modification. DNA from the commercial food products was obtained using modification I of the Sherlock kit.

The effectiveness of the modification was determined based on the parameters of DNA extracts, by assessing the parameters, such as DNA quantity (concentration) and purity (the absorbance ratio A_260/280_). For a check of the selected modification of the DNA extraction method for each sample type, four extraction repeats were performed. Then, some certain parameters, such as concentration, purity, and the working range (Wr) of repeatability, were determined. The working range of repeatability is expressed as the ratio of the amount of DNA to the weight of the sample.

Wr = (c_1 m_2)/(c_2 m_1)

where:

m_1, m_2—the weight of the first and second repeat of the sample;

c_1, c_2—the concentration of DNA obtained from each isolation repetition;

DNA from the control samples (meat and plants) was extracted using the AxFood kit without DTT (A&A Biotechnology, Gdańsk, Poland);

DNA concentration and purity were measured using a Nanodrop 2000 spectrophotometer (Thermo Fisher Scientific, Waltham, MA, USA).

### 2.2. Species and Quantitative Identification of Yellow Mealworms

The DNA extracts obtained were used to determine the following:

- The specificity of the yellow mealworm identification reaction;
- The linearity of the test for the quantitative identification of yellow mealworms;
- The limit of detection; and
- The application of the test for analysing commercial samples of food containing yellow mealworms.

#### Real-Time PCR Conditions

Each real-time PCR amplification was performed using a StepOne Plus Real-Time PCR System (Thermo Fisher, USA), with a total reaction volume of 25 μL, containing the following components: 1xTaqMan Universal Master Mix (Applied Biosystems), 340 nM of each primer (F: 5′-CAGGGTTGAACGGGTTCAGT; R: 5′-ATACTATTTCGGGCAACAGCATC), 540 nM of probe TM-Wing (6-FAMAAGCCGTACTTGTGTTACGGCGGTTCAC TAMRA (Q)) (Debode et al., 2017) [19], and 25 ng of DNA template. The real-time PCR cycling program involved a holding stage at 50 °C for 2 min and 95 °C for 10 min, followed by 42 cycles of 15 s at 95 °C and 60 s at 60 °C. A non-template control was used as a negative control in all PCR reactions. All data obtained were analysed using StepOne V.2.3 (Thermo Fisher, USA).

### 2.3. Specificity and Sensitivity Tests

The specificity of the primer pair, and the probe for mealworm identification was tested using the DNA extracted from 10 insects (yellow mealworm (adult form and larva), superworm (adult form and, larva), Dubia cockroach, Madagascar cockroach, migratory locust, lacewing, house fly, banded cricket, and field cricket), plants (lemon, banana, tomato, wheat grain and oat grain), mammals (cattle and pig), fish (trout), and birds (chicken and turkey). The PCR cross-reactions with species other than those determined should confirm the lack of species-non-specific PCR products. For all of the samples, we determined the threshold cycle (c_T_), which is correlated with the presence and original amount of biological material of which the DNA is compatible with the test primers and probe.

The presence of a PCR product for the DNA of the determined species and the concurrent lack of this product for the DNA of other species are indicative of the biological specificity of the applied test. For the c_T_ value, the absolute standard deviation (SD) and the relative standard deviation (RSD%) were determined to check the repeatability of the results obtained for the independent DNA isolations.

The sensitivity, specificity, and linearity of the real-time PCR method was evaluated using diluted DNA (25, 2.5, 0.25, and 0.025 ng) isolated from raw mealworms (separately for larvae—sample 5/1 and adult forms—sample 6/4). Dilutions were made for three independent DNA isolations. We constructed a standard curve of the above solutions and determined its slope, coefficient of correlation (R^2^) and PCR yield (%) based on the formula: E = [10 (−1/slope)—1]. Information regarding the linearity of the test was necessary to determine the potential application of the test for quantitative determinations.

### 2.4. Statistical Analysis

An analysis of the amplification c_T_ was performed for all templates. The c_T_ results are presented as standard deviation and relative standard deviation between the values. To compare the obtained c_T_ values, significant differences between both biological forms were determined using a one-way analysis of variance (ANOVA) (Excel), with a significance level of α = 0.05.

Moreover, the linearity of the test for the two biological forms of yellow mealworms was also statistically verified, using the Fisher–Snedecor test.

### 2.5. Quantitative Analysis

To visualise the applicability of the test for quantitative determinations, yellow mealworms were determined in a mixture of mealworm and superworm reference samples at ratios of 100:0, 10:90 and 1:99 (*g*/*g*) for two independent standard curves of the two templates (adults and larvae). On the basis of the obtained mealworm concentrations, the accuracy (AC%) of the method was calculated using the formula:

AC% = (c cal − c act)/(c act) × 100%

where:

c cal—calculated value of percentage concentration

c act—actual value of percentage concentration

### 2.6. Application of the Method for Commercial Food Products

To confirm the applicability of the test for analysing commercial samples of feeds that potentially contain insects, 13 processed food products containing dried mealworm larvae (*n* = 6), dried crickets (*n* = 3), freeze-dried yellow mealworms (*n* = 1), crustaceans (*n* = 2) and dipterans (*n* = 2) were analysed. Table 1 presents a detailed list of these products (type of sample—*p*).

The presence of yellow mealworms in food products was determined, based on the c_T_ value of four independent DNA isolations for each processed sample. For the obtained results, the standard deviation (SD) and relative standard deviation (RSD%) were calculated.

## 3. Results

### 3.1. DNA Extraction

For the processed samples, the DNA concentrations obtained from 0.5 g sample sizes using modification (I) were 59–408 ng/µL, with an A_260/280_ purity ratio of 1.70–1.93 (Table 2). For the raw samples of yellow mealworms and superworms, the DNA concentrations (ng/µL) and purity (A_260/280_), were 25.1–40.4 and 1.39–1.68 respectively. The first results indicate that DNA isolation from insects is not straightforward, due to the presence of chitin shells in the analysed material. Isolation from shell-free insects was easier, and standard procedure (without modification) was enough to obtain DNA of sufficient quality for further analysis. By increasing the weight of the analytical sample and by using DTT, long heating and extended vortexing (modification I), this resulted in a sufficient degree of DNA extraction for the processed samples. The steps implemented in modification (I) proved not be efficient for the raw samples (Table 2). Here, the extraction achieved from the three filtrates pooled on the filtration column from consecutive repeats of the sample (modification II) mainly improved the DNA concentration without significantly altering its purity. It is worth noting that modifications III and IV considerably improved the DNA quantity and quality. However, the DNA concentration was similar for both modifications, and a slightly better purity from a smaller sample quantity was observed for modification III. Moreover, this last type of extraction was more cost-effective, compared to modification IV. Modification III was chosen for the analysis of insects. The DNA samples obtained using this modification were characterised withthe highest purity results, and the A_260/280_ values were 1.72–1.86 for yellow mealworms and superworms. The purity of the DNA extracted from other raw insect species obtained using method III were 1.70–1.98 for most sample (Table 2).

This range was exceeded in only a few cases. The test for DNA isolation repeatability relative to the weight of the sample from the DNA (Wr) was obtained, indicating that the repeatability error was less than 28.95%. This parameter was below 20% for most of the raw samples (16 out of 20), and was outside the range for the remaining two samples (migratory locust and Dubia cockroach). The DNA concentrations obtained for the analogous samples of insects did not differ much. In most cases, they were lower than the 30% higher concentration, and only 3 (field cricket, banded cricket and superworm) out of 20 samples were significantly greater. Similarly, the differences in the purities of the obtained isolations were analogous; that is, they were less than the 20% higher purity for all samples (Table 3).

DNA extraction from the commercial food products was performed using 0.5 g sample sizes via modification I (extended heating coupled with vortexing and DTT addition). The DNA isolates were characterised with a purity of 1.70–1.93, and a DNA concentration of 60–655.9 ng/μL. Seven samples obtained utmost values of less than 100 and more than 500 (four minimal values and three maximal values). The repeatability errors for the obtained DNA concentrations that correlated with the initial weights of the sample for seven insects were below 20% (samples 2/2, 2/3, 2/4, 2/6, 2/7, 2/9, and 4/1), the next four samples (2/5, 2/8, 4/2, and 4/3) had an error of 30–50%, and the last two samples (2/1 and 4/4) had an error of above 60%. The difference in DNA concentration for both repeats of each sample was less than the 30% higher value for eight of them (2/2, 2/3, /2/4, 2/6, 2/7, 2/9, 4/1, and 4/3), 50–60% for the others (2/5, 2/8, 4/2, and 4/4) and as high as 80% for sample 2/1. The purities of the samples were always repeatable, and the difference in purity was not greater than the 20% higher value (Table 3).

Regardless of repeatability, all isolates were of sufficient quantity and quality for further analysis.

### 3.2. Yellow Mealworm DNA Detection Test

The applied real-time PCR assay allowed for the rapid (1.5 h) detection of yellow mealworm DNA. The target amplicon was short (110 bp), which increased the detection ability of yellow mealworm DNA in both the fresh template and the processed template, in which DNA was most often degraded at varying degrees.

### 3.3. Biological Specificity of the Test: Statistical Confirmation of Specificity

Positive PCR reactions were obtained for all yellow mealworm samples. No reaction products were obtained for the other species studied—field cricket, Dubia cockroach, banded cricket, superworm larvae, lacewing, house fly, lemon, banana, tomato, wheat/oat grain, cattle, pig, turkey, chicken and fish, Madagascar cockroach and migratory locust. The results of the species specificity are the c_T_ values from four independent DNA isolations of two or three independent samples of each analysedspecies (Table 4).

The mean values and standard deviation (SD) for the c_T_ in both yellow mealworm forms (12 repeats of the adult form and 12 repeats of the larval form) were 31.99 ± 3.16 and 31.61 ± 2.26 for the adult and larval forms, respectively (Table 4). The differences in the c_T_ within the same sample did not exceed 3.47 for adults and 6.52 for larvae, which translated to an RSD of 7.19 at most. The ANOVA analysis showed a lack of statistically significant differences in the c_T_ values in the yellow mealworm larval group. However, statistically significant differences were found in the c_T_ values of the adult forms (*p* < 0.05; F > Fcrit). Similarly, the ANOVA approach showed statistically significant differences in the c_T_ values between the two templates (Table 5).

The reasons of this phenomenon can be explained by differences in the body structures of both biological forms. The adult forms of mealworms have chitin shells that could have an influence on the quality and quantity of the obtained DNA and PCR products and the repeatability of the reaction.

### 3.4. Linearity and Field of the Test: Statistical Confirmation of Linearity and Specificity

We studied the standard curves plotted from the c_T_ value in the logarithm function of the DNA concentrations (25, 2.5, 0.25and 0.025) for the mealworm adults and larvae (Table 6 and Table 7). We noticed that they had R^2^ values within the range of the desirable linearity of the real-time PCR (R^2^ ≥ 0.98), as recommended by The European Network of GMO Laboratories (ENGL) for the development of real-time PCR. Similar slopes of the curves fell within the recommended range (−3.1 ≥ slope ≥ −3.6). The intercepts had higher than recommended values (27≥ intercepts ≥19). The PCR yield, calculated using the equation E = [10(−1/slope)-1], exceeded 87%. Information regarding the linearity of the test was necessary to determine the potential application of the test for quantitative determinations. The value of the intercept beyond the recommended range showed that the biological specificity of the reaction was below 100%. However, this fact wasn’t reflected in the results of the DNA cross-reactions for insects tested in this study; the DNA of other insects than yellow mealworms didn’t yield any reaction products (Table 4).

The linearity of the test for the two biological forms of yellow mealworms was also statistically verified using the Fisher–Snedecor test for the c_T_ measurements of the repeats of three independent yellow mealworm samples at dilutions of 100, 10, 1 and 0.1% (Table 7).

### 3.5. Limit of Detection

The smallest analysed amount of mealworm DNA determined the limit of determination and detection for the method (0.1%, which corresponds to an amount of 0.025 ng). For comparison, other tested insect species had comparable detection limits (house cricket [21] or lower (scuttle fly—1 pg) [22]. Obtaining a lower LOD may seem favourable for the analysis; however, note that the detection of such low concentrations had no utilitarian relevance. It is worth noting that for most commercial applications, a limit of 0.1% is sufficient.

### 3.6. Quantitative Analysis

Although all the applied curves were linear between 0.1 and 100% (Table 7), the content of the determined species calculated in the reference samples was strongly dependent on the applied calibration curve. The results obtained with four calibration curves (two curves for each type of samples) showed values of between 20 times less and 10 times more than the actual amount. The accuracies of the obtained results were approximately 10–800% (Table 8).

### 3.7. Analysis of Processed Food Containing Insects

Thirteen processed foods containing dried mealworm larvae, dried crickets, freeze-dried yellow mealworms, crustaceans and dipterans (see Table 1 for a detailed list) were selected to confirm the applicability of a real-time PCR assay presented in this study. DNA can be degraded at a high temperature and pressure, and it is important to apply this method to a variety of processed samples. Table 9 shows the results of real-time PCR for mealworm identification in the processed feed. Using a yellow mealworm-specific real-time PCR assay, we identified yellow mealworm DNA in seven products that, according to the ingredients list, contain this species. In other samples without declared yellow mealworms but containing crickets (three samples), crustaceans (two samples), and dipterans (two samples), we did not identify yellow mealworm DNA.

No reaction product was observed in samples 2/6, 2/7, and 2/9, and in the subsequent three samples (4/1, 4/3, and 4/4). In the other samples (2/1–2/5, 2/8, and 4/2), the reaction product was observed in each repeat after the 26th cycle.

The obtained results of the c_T_ values were comparable; their standard deviations did not exceed the value of 2.20, and the relative standard deviation was lower than 7.53%.

## 4. Discussion

The DNA extraction method, in which biological material is rinsed with water and DNA is extracted using the Sherlock kit/DTT/extended heating/vortexing time, is effective in obtaining DNA from raw insects. The real-time PCRs of the presented tests allowed for the rapid and efficient species identification of yellow mealworms. The method parameters point to the high sensitivity of the test, and the range of the method is 0.1–100% for mealworm content, with biological specificity in this range and linearity. The test is effective for processed products, and may be used for the verification of specific species in a food component list.

The short amplicon (<150 bp) is beneficial because it can be used to analyse food products whose DNA is often degraded to short fragments as a consequence of the influence of high temperature and pressure during the production process [23,24,25]. Nevertheless, it finds applications only in qualitative determinations, because in quantitative determinations, it is subject to excessive errors, which may undermine the reliability of the results. To the best of our knowledge, the method developed by Debode [19] is the only one available that can identify yellow mealworms. Our study shows the advantages and limitations of the test and its applicability to test, commercial food products.

We prove the suitability of the applied method for the analysis of samples that are commercially available as food for exotic animals. The availability of such a method allows us to check the credibility of the feeds offered on the market, in terms of the declared species composition. These studies may contribute to the current work on ways to control the species content of fish and non-ruminant animal feeds. In the future, the results of our research may find applications in the analogous labelling of food intended for humans.

## 5. Conclusions

Our study revealed that a test of species identification of mealworm, based on Debode primers, is an efficient method for qualitative determination in raw and processed samples. Moreover, we developed a quantitative test that, however, does not have acceptable accuracy. Using the yellow mealworm-specific real-time PCR assay, we succeeded in identifying yellow mealworm DNA in all commercial food products, which according to their ingredients lists contain this species.

## Figures and Tables

**Table 1:** Composition of test material, type of sample and code number used in the tests.

Type of Sample	Sample Composition	No. of Sample
R	field cricket	1/1
R	superworm	1/2
R	dubia cockroach	1/3
R	yellow mealworm	1/4
R	migratory locust	1/5
R	banded cricket	1/6
P	dried mealworm larvae	2/1
P	mealworm larvae 20%, dried crickets 10%	2/2
P	dried mealworm larvae	2/3
P	mealworm larvae 4%	2/4
P	mealworm 10%	2/5
P	dried cricket	2/6
P	dipterans/crustaceans	2/7
P	cricket 0.3%, mealworm 0.2%	2/8
P	crustaceans/no insects	2/9
R	mealworm	3/1
R	superworm	3/2
R	field cricket	3/3
R	migratory locust	3/4
R	madagascar cockroach	3/5
P	no insects/crustaceans	4/1
P	dried mealworm larvae	4/2
P	dipterans/crustaceans 2	4/3
P	no insects/crustaceans	4/4
R	mealworm larvae	5/1
R	superworm larva	5/2
R	field cricket	6/1
R	superworm	6/2
R	dubia cockroach	6/3
R	yellow mealworm	6/4
R	migratory locust	6/5
R	banded cricket	6/6
R	superworm larva	6/8

R—raw samples, P—processed samples (commercial samples of available pet food). Repeated samples of the same species come from different batches of insects that were purchased at a different time.

**Table 2:** Isolation parameters for consecutive DNA extraction modifications. Sample composition.

	No. of Sample	Modification							
		I	II			III		IV	
		c [ng/uL]	A_260/280_	c [ng/uL]	A_260/280_	c [ng/uL]	A_260/280_	c [ng/ul]	A_260/280_
superworm	3/2	29.8	1.70	36.4	1.40	110.9	1.84	58.0	1.78
mealworm	3/1	23.0	1.47	39.0	1.39	450.5	1.86	238.3	1.94
mealworm larva	5/1	40.4	1.64	59.9	1.70	75.0	1.72	84.2	1.70
superworm larva	5/2	25.1	1.92	62.8	1.68	185.3	1.82	127.6	1.81

I–IV—Modifications of isolations of DNA, c—concentration, A_260/280_—absorbance.

**Table 3:** Parameters of DNA isolates of insects (R) and processed commercial food products (*p*).

Type of Sample	Sample Composition	No. of Sample	c [ng/uL]	A_260/282_	m [g]	c [ng/uL]	A_260/282_	m [g]	*W*r	DNA Content (R)			Purity		
R	field cricket	3/3	554.8	1.85	0.51	671.1	1.87	0.51	0.827	116.30	<	201.33	0.02	<	0.19
	field cricket	6/1	39.5	1.61	0.45	58.4	1.39	0.55	0.827	18.90	<	17.52	0.22	<	0.16
	superworm	3/2	39.8	1.70	0.45	58.0	1.78	0.55	0.839	18.20	<	17.40	0.08	<	0.18
	superworm	6/2	154.9	2.00	0.55	110.9	1.84	0.45	1.143	44.00	<	46.47	0.16	<	0.20
	yellow mealworm	1/4	21.8	1.44	0.51	29.4	1.36	0.54	0.785	7.60	<	8.82	0.08	<	0.29
	yellow mealworm	3/1	23.0	1.45	0.50	20.7	1.43	0.50	1.111	2.30	<	6.90	0.02	<	0.15
	yellow mealworm	6/4	450.3	2.13	0.51	424.1	2.13	0.51	1.062	26.20	<	135.09	0.00	<	0.21
	migratory locust	3/4	35.5	1.54	0.49	39.3	1.59	0.48	0.884	3.85	<	11.79	0.05	<	0.16
	migratory locust	6/5	754.2	1.87	0.50	982.1	1.87	0.50	0.768	227.90	<	294.63	0.00	<	0.19
	banded cricket	1/6	221.6	1.90	0.50	262.2	1.73	0.50	0.845	40.64	<	78.67	0.17	<	0.19
	banded cricket	6/6	341.2	1.92	0.55	221.3	1.90	0.46	1.290	119.90	>	102.36	0.02	<	0.19
	Dubia cockroach	1/3	385.5	1.86	0.52	352.4	1.84	0.51	1.073	33.10	<	115.65	0.02	<	0.19
	Dubia cockroach	6/3	395.8	1.86	0.52	385.5	1.86	0.51	1.007	10.30	<	118.74	0.00	<	0.19
	Madagascar cockroach	3/5	184.7	1.79	0.47	210.3	1.85	0.54	1.009	25.60	<	63.09	0.06	<	0.19
	Madagascar cockroach	6/7	162.7	1.82	0.53	154.8	1.78	0.53	1.051	7.90	<	48.81	0.04	<	0.18
	mealworm larva	5/1	75.0	1.72	0.55	60.3	1.74	0.45	1.018	14.70	<	22.50	0.02	<	0.17
	mealworm larva	6/7	52.8	1.78	0.48	74.6	1.88	0.52	0.767	21.80	<	22.38	0.10	<	0.38
	mealworm larva	6/9	85.2	1.86	0.45	117.6	1.94	0.50	0.805	32.40	<	35.28	0.08	<	0.19
	superworm larva	5/2	185.3	1.82	0.49	141.8	1.76	0.45	1.200	43.50	<	55.59	0.06	<	0.18
	superworm larva	6/8	422.1	1.84	0.54	352.4	1.82	0.48	1.065	69.70	<	126.63	0.02	<	0.18
*p*	dried mealworm larvae	2/1	175.4	1.85	0.45	590.8	1.90	0.55	0.363	415.42	>	177.23	0.05	<	0.38
	mealworm larvae 20%, dried crickets 10%	2/2	463.6	1.77	0.45	655.9	1.80	0.52	0.817	192.26	<	196.76	0.03	<	0.36
	dried mealworm larvae	2/3	329.5	1.93	0.55	243.8	1.80	0.48	1.180	85.71	<	98.86	0.13	<	0.39
	mealworm larvae 4%	2/4	287.3	1.88	0.55	271.9	1.80	0.45	0.864	15.390	<	86.18	0.08	<	0.38
	mealworm 10%	2/5	129.8	1.80	0.45	231.0	1.80	0.54	0.674	101.180	>	69.29	0.00	<	0.36
	dried cricket	2/6	110.5	1.72	0.51	91.7	1.80	0.50	1.181	18.791	<	33.14	0.08	<	0.36
	dipterans/crustaceans	2/7	460.0	1.78	0.48	538.6	1.90	0.45	0.801	78.600	<	161.57	0.12	<	0.38
	cricket 0.3%, mealworm 0.2%	2/8	211.6	1.78	0.46	363.5	1.80	0.55	0.696	151.850	>	109.04	0.02	<	0.36
	crustaceans/no insects	2/9	408.9	1.81	0.48	420.5	1.84	0.51	1.033	11.586	<	126.14	0.03	<	0.37
	no insects/crustaceans	4/1	281.9	1.70	0.54	307.8	1.80	0.48	0.814	25.950	<	92.34	0.10	<	0.36
	dried mealworm larvae	4/2	110.3	1.79	0.48	214.8	1.88	0.51	0.546	104.500	>	64.45	0.09	<	0.38
	dipterans/crustaceans	4/3	100.7	1.73	0.52	70.9	1.79	0.49	1.338	29.770	<	30.20	0.06	<	0.36
	no insects/crustaceans	4/4	171.6	1.77	0.55	89.4	1.70	0.48	1.675	82.220	<	51.48	0.07	<	0.35

*W*r—repeatability of DNA isolations, c—concentration of DNA isolations, A_260/280_—absorbance of DNA isolations, m—weight of sample.

**Table 4:** The reaction results of yellow mealworm determination from four independent DNA isolations and interpretation of the test.

Type of Sample	Sample Composition	No. of Sample	c_T_ of DNA Isolation Repeats				SD	RSD%	Interpretation of the Result
			1	2	3	4			
R	field cricket	1/1	NR	NR	NR	NR			−
R	field cricket	3/3	NR	NR	NR	NR			−
R	superworm	1/2	NR	NR	NR	NR			−
R	superworm	3/2	NR	NR	NR	NR			−
R	Dubia cockroach	1/3	NR	NR	NR	NR			−
R	Dubia cockroach	6/3	NR	NR	NR	NR			−
R	Madagascar cockroach	3/5	NR	NR	NR	NR			−
R	Madagascar cockroach	6/7	NR	NR	NR	NR			−
R	yellow mealworm	1/4	36.38	33.92	32.91	35.25	1.52	4.39%	+
R	yellow mealworm	3/1	31.25	35.74	31.80	32.31	2.02	6.17%	+
R	yellow mealworm	6/4	30.22	26.29	30.69	27.17	2.19	7.65%	+
R	migratory locust	1/5	NR	NR	NR	NR			−
R	migratory locust	3/4	NR	NR	NR	NR			−
R	banded cricket	1/6	NR	NR	NR	NR			−
R	banded cricket	6/6	NR	NR	NR	NR			−
R	mealworm larva	5/1	33.35	28.20	31.34	33.08	2.37	7.52%	+
R	mealworm larva	6/7	28.65	30.09	31.31	33.47	2.04	6.61%	+
R	mealworm larva	6/9	31.82	29.44	32.64	35.96	2.70	8.31%	+
R	superworm larva	5/2	NR	NR	NR	NR			−
	lacewing		NR	NR	NR	NR			−
	house fly		NR	NR	NR	NR			−
	lemon		NR	NR	NR	NR			−
	banana		NR	NR	NR	NR			−
	tomato		NR	NR	NR	NR			−
	wheat/oat grain		NR	NR	NR	NR			−
	cattle		NR	NR	NR	NR			−
	pig		NR	NR	NR	NR			−
	turkey		NR	NR	NR	NR			−
	chicken		NR	NR	NR	NR			−
	fish		NR	NR	NR	NR			−

R—raw samples, NR—no reaction, c_T_—threshold cycle, SD—standard deviation, RSD%—relative standard deviation, + contains yellow mealworm, − contains no yellow mealworm.

**Table 5:** One-way analysis of variance for both forms of yellow mealworm (larva and adult form).

Template	No. of Analysed Sample Repeat	F	*p*	Fcrit	Interpretation
Mealworm larvae, sample 1	4	0.449	0.652	4.256	
Mealworm larvae, sample 2	4				
Mealworm larvae, sample 3	4				
Yellow mealworm, sample 1	4	10.218	0.005	4.256	*
Yellow mealworm, sample 2	4				
Yellow mealworm, sample 3	4				
Between both groups/templates		3.493	0.022	2.773	*

F—Fisher-Snedecor test result, *p*—probability value, Fcrit—critical value of the Fisher-Snedecor test, *—significant difference.

**Table 6:** Inter-laboratory validation results of the real-time PCR system using standard curves for three different samples—reaction results.

Actual Concentration [%] in the Reference Sample	c_T_ of Yellow Mealworms Samples Repeat			Biological Form
	1	2	3	
100	27.98	28.58	27.56	Adult (sample 6/4)
10	31.28	31.92	30.80	
1	34.91	34.95	35.70	
0.1	37.82	37.75	36.99	
100	29.32	28.37	32.57	Larva (sample 5/1)
10	32.94	31.70	35.51	
1	36.60	35.06	38.82	
0.1	39.26	38.43	40.13	

c_T_—Threshold cycle.

**Table 7:** Inter-laboratory validation results of the real-time PCR system using standard curves for three different samples—parameters of standard curves.

Parameters of Real-Time PCR	Yellow Mealworm Sample Repeat			Biological Form	F	Fcrit	Interpretation
	1	2	3				
E	103.51	108.664	100.173	Adult (sample 6/4)	2.17	19	** I
R^2^	0.986	0.987	0.998				
Slope	−3.241	−3.13	−3.318				
Y-Inter	34.183	35.051	34.668				
E	98.221	87.519	100.475	Larva (sample 5/1)	1.70	19	**
R^2^	1	1	0.986				
Slope	−3.365	−3.662	−3.311				
Y-Inter	35.062	36.0599	38.824				

E—efficiency, R^2^—coefficient of correlation, Y-Inter—the intercepts, **—the linearity of the test.

**Table 8:** Quantitative analysis of reference samples.

Type of Sample	c and c_T_ of the References Samples of Yellow Mealworm					AC [%]	
	Actual	Determined According to Curve					
		1		2		1	2
	c [%]	ct	c [%]	ct	c [%]		
Adult	100	27.85	90.22	28.33	81.16	9.78%	18.84%
	10	30.09	18.50	30.63	16.53	85.03%	65.30%
	1	31.60	6.25	32.11	5.89	525.02%	489.00%
Larva	100	31.83	9.13	28.95	959.91	90.87%	859.91%
	10	35.52	0.73	32.61	75.23	92.71%	652.34%
	1	38.43	0.06	36.27	5.90	94.18%	489.66%

c—concentration, c_T_—threshold cycle, AC—accuracy according to curve.

**Table 9:** Results of determination of yellow mealworm in commercial food products.

Type of Sample	No. of Sample	c_T_ of Samples Repeat				c_T_ Mean	SD	RSD %	Interpretation of the Result
		1	2	3	4				
P	2/1	26.65	24.35	27.04	24.52	25.64	1.40	5.47%	+
P	2/2	31.32	27.26	30.99	27.44	29.25	2.20	7.53%	+
P	2/3	31.87	31.88	31.62	31.04	31.60	0.39	1.25%	+
P	2/4	32.52	32.39	32.28	32.55	32.44	0.12	0.38%	+
P	2/5	27.21	26.38	26.95	26.56	26.78	0.38	1.40%	+
P	2/6	NR	NR	NR	NR				-
P	2/7	NR	NR	NR	NR				-
P	2/8	35.98	35.33	35.73	35.48	35.63	0.29	0.80%	+
P	2/9	NR	NR	NR	NR				-
P	4/1	NR	NR	NR	42.55				-
P	4/2	27.17	25.88	26.92	26.00	26.49	0.65	2.45%	+
P	4/3	NR	NR	NR	NR				-
P	4/4	NR	NR	NR	NR				-

*p*—processed samples, NR—no reaction, c_T_—threshold cycle, SD—standard deviation, RSD%—relative standard deviation.

## Data Availability

This paper is finish results research about identification of DNA of mealworm. All obtained results were used in this publication.
